# Risk-reducing surgery for individuals with cancer-predisposing germline pathogenic variants and no personal cancer history: a review of current UK guidelines

**DOI:** 10.1038/s41416-023-02296-w

**Published:** 2023-05-31

**Authors:** Rebecca L. McCarthy, Ellen Copson, William Tapper, Helen Bolton, Alex H. Mirnezami, J. Robert O’Neill, Nimesh N. Patel, Marc Tischkowitz, Ramsey I. Cutress

**Affiliations:** 1 University Hospital Southampton NHS Trust, Southampton, UK; 2 Faculty of Medicine, University of Southampton, Southampton, UK; 3 Cancer Sciences Academic Unit, University of Southampton, Southampton, UK; 4 University of Southampton Faculty of Medicine Health and Life Sciences, Southampton, UK; 5 Department of Gynaecological Oncology, Cambridge University Hospitals NHS Foundation Trust, Addenbrooke’s Hospital, Cambridge, Cambridgeshire UK; 6 Cambridge Oesophagogastric Centre, Addenbrooke’s Hospital, Cambridge, Cambridgeshire UK; 7 Department of Otolaryngology, University Hospital Southampton NHS Foundation Trust, Southampton, UK; 8 Department of Medical Genetics, National Institute for Health Research Cambridge Biomedical Research Centre, University of Cambridge, Cambridge, UK

**Keywords:** Surgical oncology, Cancer screening, Cancer genetics, Genetics research, Population screening

## Abstract

Identifying healthy carriers of germline pathogenic variants in high penetrance cancer susceptibility genes offers the potential for risk-reducing surgery. The NHS England National Genomic Test Directory offers germline and somatic testing to patients with certain cancers or rare and inherited diseases, or, in some cases, to their relatives. This review summarises current UK guidelines for risk-reducing surgical interventions available for individuals with no personal history of cancer, who are determined to carry germline pathogenic variants. An electronic literature search of NICE guidelines and PubMed citable articles was performed. NICE guidelines are available for bilateral mastectomy and are currently in development for risk-reducing bilateral salpingo-oophorectomy. Guidelines developed with affiliation to, or through relevant British Surgical Societies or international consensus, are available for risk-reducing hysterectomy, polypectomy, gastrectomy, and thyroidectomy. There is a disparity in the development and distribution of national guidelines for interventions amongst tumour types. Whilst we are focusing on UK guidelines, we anticipate they will be relevant much more generally and so of interest to a wider audience including where there are no national guidelines to refer to. We suggest that, as genetic testing becomes rapidly more accessible, guideline development for interventions should be more closely aligned to those for testing.

## Introduction

Germline pathogenic variants (GPVs) in cancer predisposition genes play an important role in cancer susceptibility [1, 2]. The frequency of GPVs contributing to cancer varies between cancer subtypes from 4 to 19% [1, 3]. Identifying healthy carriers of GPVs in high penetrance cancer susceptibility genes offers an opportunity for early detection or prevention of cancer. Advances in technology and completion of the 100,000 genomes project resulted in the restructuring and centralisation of the NHS Genomic Medicine Service (GMS), with the aim to promote genomic testing within mainstream medicine [4]. At present, the NHS England National Genomic Test Directory offers germline and somatic testing to patients with certain cancers where criteria are met, such as breast and pancreatic cancer, and for rare and inherited disease. When a GPV in a causal gene is identified, pre-symptomatic testing can then be offered to blood relatives [5].

The NHS GMS is at the forefront of integrating genomic testing into routine care and aims to provide equity of genomic testing, aiming to sequence 500,000 whole genomes in England by 2024 [4]. This highlights huge advances in technology with the new Illumina NovaSeq X sequencer. Over the last two decades, there has been an increase in genetic testing for cancer, for example, between 2007 and 2019 one study showed a threefold increase in germline *BRCA1/2* testing in ovarian cancer [6]. Increased somatic testing of cancers will also identify patients who may have underlying GPVs, while germline whole genome sequencing will directly identify GPVs. As testing in patients with cancer becomes mainstream it will have wider implications on relatives of those found to have deleterious variants in cancer predisposition genes and increase identification of healthy carriers of GPVs. For example, an estimated 175,000 people in the UK have Lynch syndrome (LS), however only 5% of this group are estimated to be aware of this status [7]. With increased genomic testing we anticipate identifying a larger group of cancer GPV carriers. There are several advantages to the identification of healthy carriers of GPVs including access to surveillance with potentially early detection, and prevention of cancer with risk-reducing surgical interventions. Potential disadvantages include detection of variants of unknown significance (VUS), and areas where there might be uncertainty and/or lack of clear guidance for management.

Current management options for carriers of GPVs can include active surveillance, medical, endoscopic, and surgical management. Options for medication which reduces the risk of cancer are currently limited, therefore cancer prevention in this setting is usually in the form of risk-reducing surgery (RRS).

This review focuses on summarising the current UK guidelines for risk-reducing surgical interventions available for individuals who are found to have GPVs with no personal history of cancer. It also discusses the implications of increased genomic testing on the management of people found to have GPVs.

## Methods

### Identification of germline pathogenic variants

For the purposes of this review, a GPV is defined as per the Cancer Variant Interpretation Group UK classification [8], and does not apply to benign variants or VUS.

**Fig. 1:**
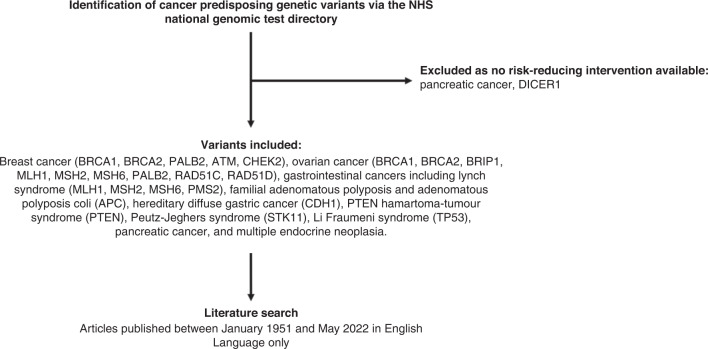
Flowchart of shortlisting used to identify of germline pathogenic variants. Variants included in the literature search were selected based upon the NHS national genomic test directory. Germline pathogenic variants without possible risk-reducing interventions were excluded.

GPVs were shortlisted for literature review based on current germline testing being offered by the NHS England National Genomic Test Directory (April 2022) [5], the UK Cancer Genetics Group (UKCGG) [9] and a summary of the USA guidelines [10]. GPVs were only included if a risk-reducing surgical intervention was available for healthy carriers. GPVs identified include those causing breast cancer (*BRCA1, BRCA2, PALB2, ATM, CHEK2*), ovarian cancer (*BRCA1, BRCA2, BRIP1, MLH1, MSH2, MSH6, PALB2, RAD51C, RAD51D, SMARCA4*), gastrointestinal cancers including Lynch syndrome (*MLH1, MSH2, MSH6, PMS2*), familial adenomatous polyposis, adenomatous polyposis coli (*APC*), MUTYH-associated polyposis (*MUTYH*), Juvenile polyposis syndrome (BMPR1A, SMAD4), hereditary diffuse gastric cancer (*CDH1*), PTEN hamartoma-tumour syndrome (*PTEN*), Peutz–Jeghers syndrome (*STK11*), Li Fraumeni syndrome (*TP53*), and multiple endocrine neoplasia type 2 (*RET*). Figure 1 summarises the identification of GPVs to be included.

### Literature search

Clinical guidelines from the National Institute for Health and Care Excellence (NICE) and Scottish Intercollegiate Guidelines Network (SIGN) were screened to identify any existing recommendations for the management of GPVs. Search terms ‘preventative’, ‘prevention’, ‘prophylaxis’, ‘prophylactic’, ‘surveillance’, ‘risk-reducing’ and ‘genetic’ were used to manually screen both guidelines and guidelines in development.

An electronic literature review was performed using PubMed to identify any journal articles summarising UK guidelines for the management of cancer-predisposing genetic syndromes. In the absence of any existing published UK management guidelines, further PubMed searches were performed to identify peer-reviewed publications outlining the management of individual hereditary cancer syndromes. All identified articles published in English language between January 1951 and May 2022 were assessed for suitability. Search terms included are detailed in supplementary table 1. Titles and abstracts of the publications identified by the PubMed searches were screened to assess suitability for inclusion.

British society guidelines were manually searched via their websites. Following the completion of the literature search, local expertise was sought to ensure no further UK guidelines were missed. Overview of literature search and outcomes is provided in Table 1.

**Table 1:** Defining the process of literature search for existing guidelines and guidelines in development.

Step	Detail	Outcome
1	Searching national genomic test directory to identify cancer-predisposing genetic variants	List of conditions and mutations which are tested for as of April 2022 [4]
2	PubMed search to identify existing guidelines which summarise the management of people with cancer-predisposing genetic variants	No UK guidelines identified. U.S.A. summary of guidelines (2006) identified which highlighted the lack of multiple endocrine neoplasia as a condition included in the national genomic test directory [9].
3	NICE guidelines search (preventative/ prevention/ prophylaxis/ prophylactic/ surveillance/risk-reducing/genetic.	Identified management of inherited breast cancer syndromes [CG164] [13] Some guidelines available for Lynch syndrome [NG151] [56]
4	NICE guidelines ‘in development’ searched	Ovarian cancer guidelines in development [38]—due for publication March 2024
5	British society guidelines searched. UK cancer genetics group (UKCGG), British Society of Gastroenterologists (BSG), Royal College of Obstetricians and Gynaecologists (RCOG), British Gynaecological Cancer Society (BGCS), British Association of Endocrine and Thyroid Surgeons (BAETS), Association of Cancer Physicians (ACP), Association of Upper GI Surgery of Great Britain and Northern Ireland (AUGIS) and British Association of Paediatric Surgeons (BPS) websites manually screened for guidelines.	UKCGG—directs to NICE guidelines for breast cancer [CG164 [13]] PALB2, PTEN, hereditary colorectal cancer and Lynch syndrome and TP53 [8] BSG/ACPGBI/UKCGG—guidelines for management of hereditary colorectal cancer [49] RCOG—risk-reducing bilateral salpingo-oophorectomy guidelines [32] BGCS—guidelines for management of uterine cancer [44] BAETS/British thyroid association—guidelines for management of MEN2 [68] No relevant guidelines found on AUGIS, BPS or ACP websites
6	PubMed search to identify and existing papers not endorsed by U.K. sub-speciality groups	See supplementary table 1
7	Subspecialty surgical expertise	Review of relevant sections of article by local expert surgeon in each sub-speciality for accuracy

### Guideline selection

For this review, we created a hierarchy of evidence-based available UK guidelines, and the highest level of evidence has been used to summarise current UK guidelines for each hereditary cancer syndrome. NICE guidelines, are linked to the commissioning of services in England, Wales, and Northern Ireland, whilst SIGN guidance applies in Scotland. These have been considered as higher ranking to other guidelines, followed by national or speciality society guidelines, non-British-society-affiliated journal articles and local guidelines or expertise, respectively. This review has been developed with input from consultants in oncology, surgery and clinical genetics. All sub-speciality guidelines have been reviewed by a surgeon of that speciality.

## Results

### Bilateral mastectomy

Breast cancer is the most common cancer in women [11]. Approximately 5% of new breast cancer diagnoses have been shown to occur in carriers of germline pathogenic variants [3]. The role of bilateral risk-reducing mastectomy (BRRM) has become widely accepted, and rates have increased in recent years, due to improved genetic testing and significant media attention with consequent increased awareness [12, 13]. Indications for bilateral mastectomy include GPVs in *BRCA1, BRCA2, PALB2*. It can also be indicated for other ‘high risk’ variants, including *PTEN, TP53, CDH1* and *STK11* [14]. BRRM is not currently indicated for breast cancer associated with neurofibromatosis type 1, except where there are additional risk factors [15].

#### Testing criteria

Testing criteria for inherited breast and ovarian cancer (*BRCA1, BRCA2, PALB2, ATM, CHEK2, RAD51C* and *RAD51D* truncating variants) are outlined in the NHS England National Genomic Test Directory which is updated twice per year (available at https://www.england.nhs.uk/publication/national-genomic-test-directories/) [16]. Testing is available for individuals with breast cancer, ovarian cancer, pancreatic cancer or prostate cancer, or unaffected relatives of deceased affected individuals with any of the aforementioned cancers, if they meet other prerequisites of testing. Guidelines utilise the pathology-adjusted Manchester Score [17] and the CanRisk tool [18–20] to calculate likelihood of a pathogenic variant being present, which is used to outline thresholds at which an individual living with cancer, a deceased individual who had cancer or an unaffected relative can be eligible for testing.

PTEN hamartoma-tumour syndrome (PHTS) is a multi-system disorder which predisposes an individual to an increased risk of breast, thyroid and renal cancers. It may also be associated with endometrial, colorectal, and skin cancer. It is caused by GPVs of the *PTEN* gene. For individuals with GPVs of the *PTEN* gene, there is a lifetime risk of developing breast cancer of between 67 and 85% [21–25]. Testing for *PTEN* GPVs is indicated in affected individuals with clinical features (such as macrocephaly) or in a deceased individual if appropriate tissue is available and no living affected individual is available for genetic testing [5].

GPVs in the *TP53* gene cause a TP53-related cancer syndrome (Li Fraumeni syndrome). It is associated with increased risk of multiple cancers including bone and soft tissue sarcomas, early-onset breast cancer, adrenocortical cancers, and malignant tumours of the central nervous system. Women have a risk of developing breast cancer of up to 85% by 60 years of age, the majority of which is early-onset breast cancer, with median age at diagnosis of 34 years [26]. Testing is available for an affected individual if they meet any one of the criteria as set out by the national test directory. Testing can also be performed in a deceased individual if appropriate tissue is available, and no living affected individual is available for genetic testing [5].

GPVs of the *CDH1* tumour suppressor gene are known to cause hereditary diffuse gastric cancer (HDGC) and hereditary lobular breast cancer (HLBC). HLBC is classified as the presence of a *CDH1* GPV in either an affected individual or a family with one or more lobular breast cancer cases, but without any diffuse gastric cancer. In those with *CDH1* GPV with a personal or familial history of diffuse gastric cancer, this is recategorized as HDGC [27]. For female carriers of *CDH1* GPV, there is an estimated 39–55% risk of developing lobular breast cancer by age 80 [27, 28]. Genetic testing is available, as per the NHS England National Genomic Test Directory, for affected individuals who meet the clinical criteria whereby at least one cancer is histologically confirmed [27]. It can alternatively be performed for a deceased individual where appropriate tissue is available and no living affected individual is available [5].

Peutz–Jeghers syndrome (PJS) is a multi-system disorder caused by a *STK11* (LKB1) GPV. It is associated with tumours of the gastrointestinal tract, female reproductive system (cervical and ovarian cancer), breast, pancreas, and biliary tract. There is a lifetime female breast cancer risk of 19.3–54% in PJS, making it borderline for being a high-risk gene especially where no family history of breast cancer [29]. Genetic testing is available for affected individuals who meet clinical criteria, or for deceased affected individuals where criteria are met, there is appropriate tissue available, and no living affected relative [5].

The NHS England National Genomic Test Directory allows testing outside of the criteria; however, it must be deemed appropriate by a specialist MDT [5].

NICE guidelines stratify breast cancer risk as ‘near population risk’, ‘moderate risk’ and ‘high risk’. These categories have slightly different definitions based upon lifetime risk from age 20 to risk between ages 40 and 50. High risk of breast cancer is defined as a lifetime risk from age 20 of greater than or equal to 30%, a risk between age 40 and 50 of greater than 8% or known carriers of *BRCA1, BRCA2* and *TP53* GPVs and carriers of *PTEN, CDH1* and *STK11* GPVs [14]. GPVs in ATM and CHEK2 confer a moderate risk of breast cancer.

#### Surgical interventions

NICE recommends that all women at high risk should be offered a discussion about BRRM. There are several prerequisites prior to this procedure taking place. All cases should be managed by a multidisciplinary team and should take into consideration individual risk factors including comorbidities, the woman’s current age and life expectancy. Women who are eligible for BRRM should have genetic counselling under the care of a specialist cancer genetic clinic including pre-operative counselling about psychosocial and sexual consequences of BRRM and directed towards support groups. BRRM should be carried out by a surgical team with specialist oncoplastic/breast reconstructive skills [14]. Breast reconstruction options, including skin and/or nipple-sparing mastectomy, should be discussed with the patient pre-operatively [30].

NICE guidelines for BRRM, do not detail the age threshold for performing risk-reducing surgery. US guidelines and Hanson et al. suggest that BRRM should be considered from age 20 for female carriers of TP53 GPVs [31]. Guidelines for the management of *CDH1* carriers state BRRM can be considered in hereditary lobular breast cancer and hereditary diffuse gastric cancer, however it is not generally recommended for those aged under 30 years or over 60 years [27]. Risk-reducing mastectomy is not currently recommended for female PJS by the European Hereditary Tumour group [29], however, as it falls under the ‘high-risk’ category in NICE guidelines, it can be considered for those with *STK11* GPVs in the UK [14].

#### Alternatives to BRRM

The current NHS England breast surveillance programme offers mammographic surveillance every 3 years to all women aged between 50 and 71 in England. For women at a higher than population risk of developing breast cancer additional surveillance is available. NICE guidelines outline breast imaging surveillance, including mammography, offered to women with a moderate- to high risk of breast cancer or those with a known *BRCA1/2* or *TP53* GPV. Annual MRI breast surveillance is available for young women (aged <50 years) with or at high risk of being a *BRCA* or *TP53* carrier who meet set criteria. The PHTS guideline development group of the GENTURIS European Reference Network suggest screening in individuals with *PTEN* GPVs should ideally be with annual MRI surveillance, however, this is not NICE guidance [21].

Medication to reduce the risk of breast cancer is available for women at moderate-to-high risk of breast cancer for up to 5 years. Tamoxifen is a selective oestrogen receptor modulator which offers long period of prevention of breast cancer following 5 years daily use [32]. It is offered as chemoprevention for premenopausal women, however, is contraindicated in individuals with a personal history of thromboembolic disease or endometrial cancer. Anastrozole is a non-steroidal aromatase inhibitor which has been proven to result in long-term prevention of breast cancer in post-menopausal women at increased risk of developing breast cancer following 5 years of regular use [33]. It is offered to post-menopausal women at high-risk and considered for women at moderate risk of breast cancer in the UK. It is contraindicated in women with osteoporosis, alternatively, raloxifene or tamoxifen can be offered [14]. The IBIS trials were not designed for germline pathogenic variant carriers therefore further longitudinal studies are required. Importantly, no effect was noted for invasive oestrogen receptor-negative breast cancer, the most common type of breast cancer in *BRCA1* female carriers [32, 33]^.^

### Bilateral salpingo-oophorectomy

Ovarian cancer can form part of an inherited cancer syndrome with or without association with breast cancer. GPVs which are associated with both ovarian and breast cancer include *BRCA1, BRCA2, RAD51C, RAD51D* and *PALB2*. Meanwhile, GPVs associated with ovarian cancer but not associated with breast cancer include those found in LS (*MLH1, MSH2, MSH6*), and *BRIP1* [34]. The European Society for Medical Oncology has published guidelines on the management of hereditary breast-ovarian cancer syndromes [35].

Small cell carcinoma of the ovary, hypercalcaemic type, is a rare and extremely aggressive subtype of ovarian cancer associated with somatic and germline deleterious variants of *SMARCA4* [36, 37]. Somatic *SMARCA4* testing is outlined in the national genomic test directory, but germline testing is not routinely available [5]. Given the prevalence of this condition, there currently is not enough evidence to make recommendations for genetic testing or the role of RRBSO in this population, and it is not outlined in the current RCOG recommendations. There is a strong argument for early RRBSO given the early-onset and aggressive nature of this condition [36].

#### Testing criteria

The NHS England National genomic test directory outlines testing that is appropriate for the affected individual if they have high-grade non-mucinous epithelial ovarian cancer (EOC) at any age or EOC with a family history of EOC (at least one first or second-degree relative or greater than two second- or third-degree relatives). Testing can also be performed on a deceased affected individual if appropriate tissue is available and no affected living relative is available for testing. In inherited ovarian cancer which is associated with breast cancer, this is extended to living unaffected individuals in certain circumstances [5].

#### Surgical interventions

Risk-reducing bilateral salpingo-oophorectomy (RRBSO) is the gold standard for the prevention of ovarian cancer with an 80–96% risk reduction in patients with *BRCA1/2* GPVs [38]. RRBSO does not reduce the risk of developing primary peritoneal carcinoma [38–40]. Concomitant hysterectomy is justified in LS due to increased risk of endometrial cancer, however, it may also be appropriate for a small number of women with other GPVs undergoing RRBSO for additional gynaecological indications such as fibroids or other benign conditions [34].

The Royal College of Obstetrics and Gynaecology (RCOG) has produced a summary of guidelines in conjunction with the British Journal of Obstetrics and Gynaecology regarding the role of RRBSO in individuals below the age of natural menopause [34]. RRBSO can be offered to women with moderate-to-high-risk GPVs. Moderate risk is classified a 4–10%, while high risk is classified as greater than 10% lifetime risk of developing ovarian cancer [41]. GPVs are associated with varying estimated lifetime risk of developing ovarian cancer: *BRCA1* 44% (95% CI, 36–53%), *BRCA2* 17% (11–25%) [42], *RAD51C* 11% (6–21%), *RAD51D* 13% (7–23%) [43], *MLH1, MSH2* and *MSH6* (LS) 11% (7.4–19.7%), 17.4% (11.8–31.2%) and 10.8% (3.7–38.6%), respectively [34], *PALB2* ~5% (2–10%) and *BRIP1* 5.8% (3.6–9.1%) [34, 44]. The recommended timing of RRBSO varies dependent on the risk. The timing of RRBSO is individualised, and is based upon the risk of the GPV, fertility considerations and personal preferences. RRBSO can be considered between ages 35 and 40 years for carriers of *BRCA1* GPVs and those with LS, between 40 and 45 years for those with *BRCA2* GPVs, 40 and 50 years for *RAD51C* or *RAD51D* carriers and delayed to 45 and 50 years for carriers of *BRIP1* or *PALB2* [34].

#### Special requirements

RRBSO is usually undertaken once a woman’s family is complete, although this is considered in the context of her personal circumstances and risk. It is possible to consider the option of fertility preservation with IVF or oocyte freezing, as embryos can be implanted after RRBSO to enable the woman the potential option of completing her family later. For many women RRBSO is performed prior to the age of the natural menopause, resulting in an iatrogenic ‘surgical’ menopause. Consequently, immediate menopausal symptoms are often experienced which may include vasomotor symptoms, mood changes, sleep disturbance, vaginal dryness, and sexual dysfunction. Longer-term consequences of an early menopause include increased risk of cardiovascular disease, osteoporosis, and neurocognitive effects. Hormone replacement therapy (HRT) up to 51 years of age is recommended, in the absence of any contraindication including personal history of breast cancer or venous thromboembolism. It can result in symptom relief and minimises the long-term effect of early menopause; however, it may not completely ameliorate the effects of surgery on sexual function [34]. The impact of the menopause, and the option of HRT should be discussed prior to surgery and commenced immediately after RRBSO. For women who are not having a hysterectomy, oestrogen must be used in combination with a progestogen to protect against endometrial cancer. The progestogen can be delivered directly into the uterus, with fewer adverse effects than systemic progestogen. The intra-uterine system can be placed at the time of RRBSO provided this has been discussed and agreed with the woman during the pre-operative consultation. NICE guidance recommends that HRT usage should be confined to women younger than the age of the expected natural menopause if at moderate or high risk of breast cancer [14]. Continuing beyond this will require a discussion and consideration of BRRM status.

#### Alternatives to RRBSO

The combined oral contraceptive pill (COCP) is a strong protective factor for ovarian cancer in the general population and has been demonstrated to substantially reduce the risk of ovarian cancer in women with GPVs in *BRCA1/2* GPVs (compared with less than 5 years COCP, >10 years COCP, HR 0.37, 95% CI 0.19–0.73) [45, 46]. Women should be counselled on the use of COCP and small increased risk of breast cancer [45]. There is currently no effective tool which has been developed for population-wide surveillance for the early detection of ovarian cancer. NICE guidelines for the identification and management familial and genetic risk of ovarian cancer are currently in development and due for publication March 2024 [47].

### Hysterectomy

Lynch syndrome (LS) is associated with GPVs in DNA mismatch repair (MMR) genes including GPVs of *MLH1, MSH2, MSH6, PMS2* [8, 48]. GPVs in these genes are associated with different risks for cancers including colorectal, small bowel, endometrial, ovarian, and pancreatic cancers. In women with LS, gynaecological cancers are twice as common as gastrointestinal cancers [49].

#### Testing criteria

In the UK, all new diagnoses of endometrial and colorectal cancer are eligible for tumour immunohistochemistry to identify MMR-deficient or microsatellite instability tumours that may be suggestive of LS. Germline testing can subsequently confirm the presence of GPVs in LS genes. Germline testing is also appropriate for affected individuals or unaffected individuals with family history of LS-related cancer where no affected living individual is available for testing [5].

The risk of endometrial cancer in LS varies as the genes associated with LS have different penetrance. Cumulative incidence of endometrial cancer at 75 years of age is 37% in *MLH1*, 48.9% in *MSH2*, 41.1% in *MSH6* and 12.8% in *PMS2* mutation carriers [48, 50].

#### Surgical interventions

Risk-reducing hysterectomy (RRH), often performed with bilateral salpingo-oophorectomy (BSO), is an option for patients with LS. RRH at age 40 years has been demonstrated to prevent endometrial cancer by age 50 years in carriers of *MLH1 (13%), MSH2 (16%)|* and *MSH6* (11%) GPVs who would have been expected to develop endometrial cancer [49]. Current guidelines, from the Manchester International Consensus Group, recommends that all women at risk of LS should be offered RRH with BSO at “a time appropriate to them”. This is recommended at no earlier than age 35–40 years, following completion of childbearing, and suggested from around age 35 years for carriers of GPVs of *MSH2* or *MLH1* and after age 40 years for *MSH6*. There is insufficient evidence for recommending risk-reducing gynaecological surgery for carriers of *PMS2* GPVs, however patient representatives have expressed that they should be offered RRS [51]. The British Gynaecological Cancer Society (BGCS) Uterine Cancer Guidelines support offering surgery to these women after the age of 50, as their risk prior to menopause is low [52]. The BGCS recommend post-operative HRT to women following premenopausal oophorectomy for women with LS, due to its protective effect on colorectal cancer risk [52]. For women with *MSH6* GPVs, the risk of developing ovarian cancer only starts to increase in the post-menopausal years, in contrast to an earlier onset risk for endometrial cancer. In these women, a two-stage RRS approach may be adopted, after counselling, with RRH offered from 40 years, and delayed RRSBO until after 50 years, in circumstances where the woman wishes to avoid surgical menopause and prefers to avoid taking HRT.

Recently, a few studies have reported an increased risk of serous endometrial cancer in *BRCA1* carriers estimated as 3% lifetime risk [53, 54]. This is not currently an indication of RRS [51].

#### Alternatives to RRH

There is no population-wide surveillance tool for endometrial cancer in the UK. Current international guidelines do not recommend invasive screening for carriers of MMR GPVs, however there is a recommendation that they may wish to consider the option of annual clinician review from the age of 25 to discuss red flag features of endometrial and ovarian cancer [51]. The BGCS guidelines recommend that women with LS could be offered annual surveillance with a transvaginal ultrasound scan (TVS), hysteroscopy and/or endometrial sampling from the age of 35 years after counselling about the risks, benefits and limitations of surveillance, acknowledging that there is no current high-quality evidence that surveillance improves outcomes [52]. Surveillance is not a substitute for risk-reducing surgery, but is considered an option for women who have yet to complete their families. Aspirin has been proven to reduce the risk of colorectal cancer in the LS population, however there is little evidence to support this for reducing endometrial cancer risk [55].

There is strong evidence to show that obesity is associated with a significantly increased risk of endometrial cancer in the general population, whilst use of progestogen-containing hormonal contraceptives, oral or intra-uterine, is potentially associated with a decreased risk [52]. The BGCS guidelines fall short of recommending their use as a risk reduction strategy for women with LS but acknowledge that further studies are warranted. Women with LS can be advised that maintaining a healthy weight and using progestogen-containing contraceptives may reduce their risk, although LS-specific evidence is not yet available.

### Polypectomy and colectomy

Several syndromes are associated with a predisposition to colorectal cancer. This includes LS, APC-associated polyposis, Peutz–Jeghers Syndrome (PJS), Juvenile Polyposis Syndrome (JPS) and MUTYH-associated polyposis. The British Society of Gastroenterology (BSG), Association of Coloproctology of Great Britain and Ireland (ACPGBI), and the UKCGG have produced extensive guidelines developed in accordance with the BSG NICE-compliant guideline process regarding the management of hereditary colorectal cancer [56]. They recommend colonoscopy as the gold-standard diagnostic and preventative method of surveillance for people with a hereditary risk of colorectal cancer [56]. This can guide the timing of and type of risk-reducing surgery if required.

#### Testing criteria

LS is the most common heritable cause of colorectal cancer and is associated with 10-48% cumulative risk of developing colorectal cancer by the age of 75 years dependent on the mutated MMR gene (*MLH*1 48.3%, *MSH2* 46.6%, *MSH6* 20.3%, *PMS2* 10.4%) [48]. In the UK, all new diagnoses of colorectal cancer are eligible for tumour immunohistochemistry to identify MMR-deficient tumours. Germline testing is also appropriate for affected individuals or unaffected individuals with family history of LS-related cancer where no affected living individual is available for testing [5].

Familial adenomatous polyposis (FAP) is defined by the presence of GPVs in the APC gene which predisposes an individual to colorectal and upper GI polyposis. The lifetime risk of malignancy in FAP is 100% [57]. Genetic testing for FAP is available for children or young adults who may not have developed bowel polyps but have the presence of one of the APC-associated clinical features outlined by the NHS England National Genomic Test Directory [5].

PJS is a rare autosomal dominant condition which causes hamartomas polyps and mucocutaneous pigmentation. It is caused by a GPV in *STK11* and is associated with an increased risk of multiple tumours including a 28–34% cumulative risk of developing colorectal cancer by age 64 years [58, 59]. Testing is available for the affected individual if they meet the clinical criteria, or for the deceased affected individual where appropriate tissue is available, and no living affected individual is available for testing [5].

Juvenile polyposis syndrome (JPS) is an autosomal dominant condition associated GPVs in *SMAD4* or *BMPR1A* [56]. It is characterised by hamartomatous polyps in the GI tract and one study suggested a 39% cumulative lifetime risk of colorectal cancer [60]. MUTYH-associated polyposis (MAP) is caused by GPVs of the *MUTYH* gene and is recessively inherited. It is associated with a 63% cumulative lifetime risk of colorectal cancer at age 60 years [61].

#### Surgical interventions

Most interventions for carriers of GPVs which predispose to colorectal cancer involve endoscopic surveillance. However, in some cases, preventative procedures are recommended.

In FAP, colonoscopic surveillance is recommended every one to 3 years, commencing from age 12–14 years. Risk-reducing colectomy is an option for patients with FAP. This can be through a total colectomy and subsequent ileorectal anastomosis; or a proctocolectomy with ileal pouch-anal anastomosis; or a proctocolectomy with ileostomy formation. Relative indications for preventative surgical intervention include polyps >10 mm in diameter, high-grade dysplasia within polyps and a significant increase in polyp burden between surveillance examinations [56]. The BSG/ACPGBI do not suggest an age threshold for preventative surgery, instead suggesting the time of risk-reducing surgery should be based on the risk of cancer estimated through colonoscopy and should be suitable to the patient, considering educational, social, family planning and emotional development as well as their likelihood of being compliant with follow-up surveillance testing [56]. It is important to bear in mind that risk-reducing pan proctocolectomy and ileal pouch-anal anastomosis does not completely eradicate the risk of future cancer in the pouch, as due to the techniques currently employed in the surgical construction of a pouch, a small amount of residual rectal mucosa and or the anal transition zone mucosa are preserved, which may lead to a future cancer risk [62]. As a result, annual endoscopic surveillance of the pouch is recommended for life [56].

#### Surgical interventions

In the future, patients with LS may be referred to the NHS Bowel Cancer Screening Programme [7]. In JPS 1–3 yearly surveillance should commence from age 15 years (or earlier if symptomatic). A small minority of very rare conditions which predispose to colorectal cancer (e.g., polymerase proofreading associated polyposis or NTHL1 associated polyposis), the BSG/ACPGBI/UKCGG state that due to insufficient clinical data to develop specific guidance, these cases should be managed in a multidisciplinary expert centre [56].

#### Medical interventions

Both NICE and the BSG/ACPGBI/UKCGG recommend daily aspirin to reduce the incidence of LS-related colorectal cancer [56, 63, 64].

### Gastrectomy

Hereditary diffuse gastric cancer (HDGC) is an autosomal dominant inherited condition characterised by a high prevalence of early-onset diffuse-type gastric cancer (DGC) and lobular breast cancer. Recent guidelines from the international gastric cancer linkage consortium (IGCLC) have revised the definition of HDGC to the presence of a *CDH1* or *CTNNA1* GPV in either an individual with DGC, or unaffected individual with a family pedigree featuring one or more DGC cases in first-degree or second-degree relatives, with or without a personal or family history of lobular breast cancer [27]. The cumulative lifetime risk of developing DGC is estimated to be between 42 and 70% in men with HDGC, and 33 and 56% in women with HDGC [28, 65].

#### Testing criteria

Genetic testing for *CDH1* GPVs, and soon to include CTNNA1 GPVs, is available for affected individuals who meet the set clinical criteria whereby at least one cancer is histologically confirmed. It can alternatively be performed for a deceased individual where appropriate tissue is available, and no living affected individual is available. Full criteria are detailed in the NHS England National Genomic Test Directory [5]. The IGCLC suggest *CTNNA1* testing should be considered for individuals who meet the criteria for genetic testing and no *CDH1* GPVs are identified [27].

#### Surgical interventions

A recommendation for prophylactic total gastrectomy (PTG) is made for all carriers of *CDH1* GPVs with a family history of DGC. For carriers of *CDH1* GPVs without a family history of DGC, or with a family history of lobular breast cancer only, PTG should still be considered [27]. PTG is the only current effective strategy to prevent gastric cancer in patients with HDGC [66]. Surgery should ideally be undertaken between 20 and 30 years of age when the risk and benefit are most favourable and is not generally recommended for patients older than 70 years [27].

An overriding principle in PTG is to minimise risk to the patient. Choice of surgical approach should be guided by that least likely to incur a complication as a serious complication following PTG may impact not only the patient but other family members and patients with HDGC in their decision to come forward for surgery. Both minimally invasive and open approaches are well-described with one of the largest series reporting a median one-day improvement in post-operative length of stay with a laparoscopic approach [67]. As the aim of surgery is to eradicate all gastric mucosa, there should be intra-operative confirmation of oesophageal squamous mucosa in the proximal margin and duodenal mucosa in the distal margin. Lymph node metastases are rare in PTG and subsequently a D1 (perigastric) lymphadenectomy is considered a pragmatic compromise between reducing morbidity and providing adequate staging in the unexpected case an incident pathological T2 signet ring cell carcinoma is resected [68].

#### Special requirements

As signet ring cell foci are multifocal and can occur throughout the stomach in HDGC there is no role for endoscopic or limited gastric resection in the treatment of HDGC. Total gastrectomy offers the potential for cure but has several risks and long-term sequalae, therefore it is recommended that patients have pre-operative psychosocial counselling and assessment for comorbid mental illness, particularly eating disorders and addiction [69]. Patients must have a baseline endoscopy to ensure an established gastric cancer is not present prior to prophylactic gastrectomy, as this would require full staging with consideration of neoadjuvant treatment prior to surgical intervention [27]. During baseline endoscopy a detailed mucosal evaluation should be undertaken together with targeted and random biopsies following the Cambridge protocol [70].

There are several common consequences of PTG including weight loss, nutritional deficiencies, a risk of dumping syndrome, pancreatic insufficiency, small-intestinal bacterial overgrowth, and hypoglycaemia. Life-long follow-up is needed to monitor for and treat these conditions to reduce their impact on quality of life [71].

#### Alternatives to prophylactic gastrectomy

Due to the inherent risks of total gastrectomy and the significant lifestyle and nutritional consequences, many patients wish to defer surgery [72]. All patients should undergo a baseline endoscopy following diagnosis. If no lesions are identified then annual endoscopic surveillance as an alternative to PTG has been demonstrated to be safe in a specialist HDGC referral centre [73]. If signet ring cell lesions are identified during surveillance, PTG is recommended at that point [73].

### Thyroidectomy

Multiple endocrine neoplasia type 2 (MEN2) is a multi-system disease associated with GPVs of the *RET* gene and has an autosomal dominant inheritance pattern. MEN2 is subcategorised into MEN2a and MEN2b (also referred to as MEN3). Both MEN2A/B are associated with medullary thyroid carcinoma. The risk of medullary thyroid cancer is 95% in MEN2a and 100% in MEN2b [74]. MEN2b accounts for ~5% of all MEN2 cases [74] and is associated with an earlier onset and more severe phenotype. The British Thyroid Association has produced guidelines which include the role of risk-reducing surgery in MEN2, which is adapted from the American Thyroid Association guidelines [75, 76]. In addition, *PTEN* GPVs are associated with a 35% lifetime risk of thyroid cancer development [77].

#### Testing criteria

Testing for MEN2 is outlined in the NHS England National Genomic Test Directory [5]. In total, 25% of all medullary thyroid carcinoma is familial [76], therefore testing is available for all an affected individuals with medullary thyroid carcinoma or if an individual has MEN2-related endocrine abnormalities [5]. The ATA has classified *RET* GPVs from those associated with the highest to moderate risk of medullary thyroid carcinoma [75]. Prenatal testing is possible to screen for *RET* GPVs with specialist input from clinical genetics [76].

Testing for *PTEN* GPVs is indicated in affected individuals with clinical features (such as macrocephaly) or in a deceased individual if appropriate tissue is available and no living affected individual is available for genetic testing [5].

#### Surgical interventions

Risk-reducing thyroidectomy is an option for carriers of *RET* GPVs and has dramatically improved outcomes in patients with MEN2. Risk-reducing surgery should be offered to disease-free carriers of RET GPVs. Given that risk-reducing surgery is an option for children, this should be discussed with parents before testing a child for a *RET* GPV. Timing of risk-reducing surgery is dependent on the risk of the variant such that children with the highest-risk variant, c.2753 T > C (p.Met918Thr), would be recommended to have risk-reducing surgery within the first year of life and those with high-risk *RET* GPVs (codon 634 changes) are recommended to have risk-reducing surgery before age five Meanwhile, children with moderate risk *RET* GPVs may delay surgery to beyond 5 years of age [75, 76], and the common 2410 G > A p.(Val804Met) variant appears to be associated with a low risk [78].

#### Alternatives to surgery

Ultrasound neck examination and calcitonin level monitoring are possible and should be introduced in the first year of life for highest-risk RET GPV carriers, or after age 3–5 years for all other *RET* GPV carriers [74, 76]. The UKCGG recommends a minimum of annual ultrasound thyroid surveillance from age 16 years, dependent on family history, for carriers for *PTEN* GPVs [77]. Risk-reducing thyroidectomy can be considered [79].

## Discussion

**Fig. 2:**
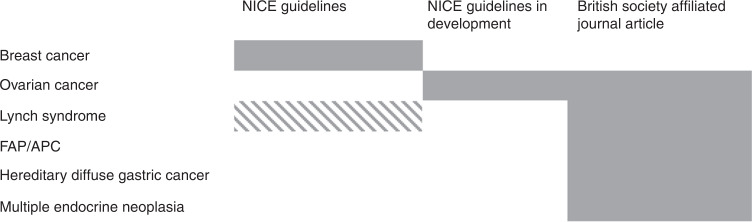
Summary of guidance for each tumour type. The level of guidance available by tumour type. A solid block indicates full guidance available, while a striped block illustrates where partial guidance is available. Breast cancer is the only tumour type which has full NICE-guidelines. Lynch syndrome is partially covered in NICE guidelines, while ovarian cancer has NICE guidelines in development. All other tumour types guidelines are currently available in journal articles.

This review summarises the current genomic indications for RRS, including mastectomy, bilateral salpingo-oophorectomy, hysterectomy, gastrectomy, polypectomy, and thyroidectomy, as well as highlighting surveillance available for healthy carriers of GPVs. The guidelines vary from NICE guidelines to recommendations by British specialist societies from international consensus guidelines to individual journal article recommendations. Figure 2 summarises the level of guideline available for each tumour type. It is evident that there is inconsistency in the level of guidelines available for each tumour type. Guidance for the management of healthy carriers of GPVs, and surgical management, may not be keeping pace with the developments and advances in testing. NICE guidelines take several years to develop, meanwhile the NHS England National Genomic Test Directory is updated twice per year. The most recent NHS England National Genomic Test Directory should be referred to for most up-to-date eligibility criteria for testing. Guideline development for testing should be more closely linked with development and guidance for intervention and management strategies.

The rates of RRS for *BRCA1/2* carriers have increased over the last two decades [80]. However, the age at which to perform RRS remains controversial in many cancer types. For example, in breast cancer, surveillance can begin at age 25 years for high-risk carriers, however there is no age-specific guidance for the timing of BRRM. As testing increases and guidelines become more sophisticated, timing of RRS needs to be considered, taking risk, cost-effectiveness, and available surveillance strategies into consideration [41].

Historically, there has been disparity in the availability of testing across the UK. There are differences in the way in which genomics has become integrated into healthcare across the four nations and the differences in the NHS structure and systems [4]. To the authors’ knowledge, there are currently no specific Scottish Intercollegiate Guidelines Network (SIGN) guidelines relating to the role of RRS in Scotland. Genome UK has set out a strategy to implement genomic testing across the four nations and provide equity of testing across the UK [81]. However, funding for intervention also varies across the UK. Due to the lack of NICE guidelines, clinical commissioning groups are responsible for funding interventions, such as RRS. This will likely lead to inequal access to risk-reducing interventions for patients.

The guidelines for intervention outlined in this review are based upon the presence of a specific GPVs, designated as pathogenic and according to the NHS England National Genomic Test Directory. VUS are variants detected during sequencing, for which there is insufficient evidence for or against pathogenicity [82]. In a retrospective cohort study of individuals who had genetic testing at a single laboratory, over a 10-year period, 24.9% of all reported variants of uncertain significance were reclassified. 97.0% of all reported reclassified VUS were downgraded [83]. Clinical decisions cannot be made on the presence of VUS, however, over time these variants may be reclassified as pathogenic or non-pathogenic, with the majority downgraded [8, 83]. As a result, there is a possibility of healthy people undergoing RRS or invasive surveillance unnecessarily, being exposed to unnecessary harm [84], and anecdotally this has become the subject of a number of medico-legal cases. Conversely, there is a possibility that VUS are reclassified as pathogenic [83]. This could also result in patient harm if a person develops a cancer which could have been detected through earlier surveillance or prevented through RRS [8].

The Cancer Variant Interpretation Group UK (CanVIG) has developed a detailed framework [8] regarding clinical actions after reclassification. This needs to be taken into consideration when performing genetic testing and when advising patients on preventative or surveillance interventions.

VUS and variant reclassification pose a challenge for communication with patients, and a significant problem for clinicians and healthcare systems. CanVIG recently published a framework how to discuss the presence of VUS with patients [8]. The UK Joint Committee of Genomics in Medicine guidance recommends that patients should be informed on how they might be recontacted by a service or how they can seek an update [8, 82, 83, 85]. Formalised, national systems need to be in place to reduce the chance of patient coming to harm through reclassification of a VUS not being communicated.

Ethical considerations are important when communicating with patients. Surgical prophylaxis is controversial. In most inherited cancer syndromes, the patient will not definitely develop cancer, while surgery is not always guaranteed to prevent the development of cancer and can have life-changing implications. There must be careful consideration of the risk of developing cancer balanced against the effectiveness of a surveillance programme and the morbidity of surgical intervention and potential alternative management strategies. Effective communication is vital when discussing risk with healthy individuals to enable them to make an informed decision. Genomic medicine services often provide letters or leaflets for affected patients carrying GPVs to share with relatives whom it may affect. However, on rare occasions, known carriers may refuse to share information with at-risk relatives, which is ethically challenging. The joint committee on Genomics in Medicine outlines how to approach these situations and, in line with GMC guidance, when it is appropriate to break confidentiality and how to disclose to affected relatives without disclosing confidential information [86]. With the integration of genomic testing into routine clinical care, there will be a need for increased access to trained genetic counsellors to support patients through this decision-making.

The UK is at the forefront of integrating genomic testing into routine clinical care [81]. Many challenges will be faced with increased genomic testing including in creating and reviewing guidelines for testing, interpretation, communication of results and intervention. Next-generation sequencing (NGS) is now available worldwide, but there are few data published on the implementation of NGS into clinical care globally [87] and how to manage and integrate results into routine clinical care. In addition, increased demand on clinical genetics and surgical services will inevitably result in workforce capacity issues. With improved technology and testing, increased funding of clinical services will be required to ensure results are interpreted and acted upon, and patients are provided with the opportunities to discuss and receive risk-reducing surgical interventions or surveillance where appropriate.

## Conclusion

This review summarises the current indications for risk-reducing surgical procedures for healthy carriers of GPVs. There is currently a disparity in the development and distribution of national guidelines for interventions across tumour sites. For example, there are well-established but outdated NICE guidelines on the role of BRRM, but little guidance on the role of RRS in MEN2. There is a rapid progression of genomics in cancer medicine, however there is a risk of clinicians and patients receiving results without clear guidance for possible interventions, resulting in the potential for variation in and the possibility of inappropriate patient management. We suggest as testing becomes rapidly more accessible, guideline development for intervention needs to be more closely aligned to those of testing.

## Supplementary information

Supplementary material
