# Supplementary material for: Risk-reducing surgery for individuals with cancer-predisposing germline pathogenic variants and no personal cancer history: a review of current UK guidelines
Source: Br J Cancer. 2023 May 31;129(3):383–92. doi: 10.1038/s41416-023-02296-w (PMC10403612; doi:10.1038/s41416-023-02296-w)
Supplement: Supplementary file 1 — Supplementary material [file 41416_2023_2296_MOESM1_ESM.docx]

Supplementary material for the article titled:

**Risk-reducing surgery for individuals with cancer-predisposing germline pathogenic variants and no personal cancer history: A review of current UK guidelines.**

**Authors:** Rebecca L. McCarthy^1,2^, Ellen Copson^1,3^, William Tapper^4^, Helen Bolton^5^, Alex H. Mirnezami^1,3^, J. Robert O’Neill^6^, Nimesh N. Patel^7^, Marc Tischkowitz^8^, Ramsey I. Cutress^1,3^

Supplementary material (Supplementary Table 1) outlining PubMed search terms used in data collection to identify existing guidelines for risk-reducing surgery in patients with cancer predisposing genetic variants and no personal cancer history.

| Supplementary Table 1: PubMed search terms used. Results were manually screened by title and subsequently by abstract to identify appropriate guidelines for review. | | | |
| --- | --- | --- | --- |
| Condition |  | Search terms | Results |
| All cancer predisposing genetic variant conditions | 1 | ((((Prophylactic [Title/Abstract]) OR (Risk-reducing [Title/Abstract])) AND (Surgery [Title/Abstract])) AND (humans [MeSH Terms])) AND (Cancer [Title/Abstract]) | 2781 |
|  | 2 | ((((Prophylactic[Title/Abstract]) OR (Risk-reducing[Title/Abstract])) AND (Surgery[Title/Abstract])) AND (humans[MeSH Terms]) AND (Cancer[title/abstract]) AND (Guidelines[title])) | 39 |
| Breast cancer | 3 | (((((Prophylactic [Title/Abstract]) OR (Risk-reducing [Title/Abstract])) AND (Surgery [Title/Abstract])) AND (humans [MeSH Terms])) AND (Cancer [Title/Abstract])) AND (breast[Title/Abstract]) | 1031 |
| Ovarian cancer | 4 | (((((Prophylactic [Title/Abstract]) OR (Risk-reducing [Title/Abstract])) AND (Surgery [Title/Abstract])) AND (humans [MeSH Terms])) AND (Cancer [Title/Abstract])) AND ((ovarian[Title/Abstract] OR (ovary[Title/Abstract])) | 679 |
| Colorectal cancer/ Lynch syndrome | 5 | (((((Prophylactic [Title/Abstract]) OR (Risk-reducing [Title/Abstract])) AND (Surgery [Title/Abstract])) AND (humans [MeSH Terms])) AND (Cancer [Title/Abstract])) AND ((Lynch[Title/Abstract] OR (colorectal[Title/Abstract])) | 343 |
| Gastric cancer | 6 | (((((Prophylactic [Title/Abstract]) OR (Risk-reducing [Title/Abstract])) AND (Surgery [Title/Abstract])) AND (humans [MeSH Terms])) AND (Cancer [Title/Abstract])) AND (CDH1[Title/Abstract]) | 42 |
| Multiple endocrine neoplasia/thyroid cancer | 7 | (((((Prophylactic [Title/Abstract]) OR (Risk-reducing [Title/Abstract])) AND (Surgery [Title/Abstract])) AND (humans [MeSH Terms])) AND (Cancer [Title/Abstract])) AND (("multiple endocrine neoplasia"[Title/Abstract] OR (thyroid[Title/Abstract])) | 203 |
| PTEN hamartoma-tumour syndrome | 8 | (((((Prophylactic [Title/Abstract]) OR (Risk-reducing [Title/Abstract])) AND (Surgery [Title/Abstract])) AND (humans [MeSH Terms])) AND (Cancer [Title/Abstract])) AND (PTEN[Title/Abstract]) | 12 |
| Li Fraumeni syndrome | 9 | (((((Prophylactic [Title/Abstract]) OR (Risk-reducing [Title/Abstract])) AND (Surgery [Title/Abstract])) AND (humans [MeSH Terms])) AND (Cancer [Title/Abstract])) AND ('Li Fraumeni'[Title/Abstract] OR (TP53[Title/Abstract])) | 22 |
